# KLF4-mediated upregulation of the NKG2D ligand MICA in acute myeloid leukemia: a novel therapeutic target identified by enChIP

**DOI:** 10.1186/s12964-023-01118-z

**Published:** 2023-05-04

**Authors:** Reem Alkhayer, Viviane Ponath, Miriam Frech, Till Adhikary, Johannes Graumann, Andreas Neubauer, Elke Pogge von Strandmann

**Affiliations:** 1 Institute for Tumor Immunology, Center for Tumor Biology and Immunology, Philipps University of Marburg, Marburg, Germany; 2 Clinic for Hematology, Oncology, and Immunology, Center for Tumor Biology and Immunology, Philipps University of Marburg, Marburg, Germany; 3 Institute for Molecular Biology and Tumor Research, Institute for Medical Bioinformatics and Biostatistics, Center for Tumor Biology and Immunology, Philipps University of Marburg, Marburg, Germany; 4 Institute of Translational Proteomics, Philipps University of Marburg, Marburg, Germany

**Keywords:** Natural killer cells, NKG2D/NKG2D-ligand axis, Immune evasion, APTO253, Immunotherapy

## Abstract

The immunoreceptor NKG2D, which is expressed on NK cells and T cell subsets is critically involved in tumor immune surveillance. This applies in particular to acute myeloid leukemia (AML), which evades immune detection by downregulation of NKG2D ligands (NKG2D-L), including MICA. The absence of NKG2D-L on AML cells is moreover associated with leukemia stem cell characteristics. The NKG2D/NKG2D-L system thus qualifies as an interesting and promising therapeutic target.

Here we aimed to identify transcription factors susceptible to pharmacological stimulation resulting in the expression of the NKG2D-L MICA in AML cells to restore anti-tumor activity. Using a CRISPR-based engineered ChIP (enChIP) assay for the *MICA* promoter region and readout by mass spectrometry-based proteomics, we identified the transcription factor krüppel-like factor 4 (KLF4) as associated with the promoter. We demonstrated that the *MICA* promoter comprises functional binding sites for KLF4 and genetic as well as pharmacological gain- and loss-of-function experiments revealed inducible MICA expression to be mediated by KLF4.

Furthermore, induction in AML cells was achieved with the small compound APTO253, a KLF4 activator, which also inhibits MYC expression and causes DNA damage. This induction in turn yielded increased expression and cell surface presentation of MICA, thus rendering AML cells more susceptible to NK cell-mediated killing. These data unravel a novel link between APTO253 and the innate anti-tumor immune response providing a rationale for targeting AML cells via APTO253-dependent KFL4/MICA induction to allow elimination by endogenous or transplanted NK and T cells in vivo.

Video Abstract

**Supplementary Information:**

The online version contains supplementary material available at 10.1186/s12964-023-01118-z.

## Background

NKG2D (encoded by the killer cell lectin-like receptor subfamily K, member 1, or *KLRK1* gene) is a C-type lectin receptor and a major activating immunoreceptor involved in tumor immune surveillance. NKG2D is expressed on the surface of most cytotoxic lymphocytes such as Natural killer (NK) cells, CD8+ T cells, some γδ T cells, and possibly also on some CD4+ T cells and is known as a sensor for damaged or dangerous cells [1, 2]. Ligands for NKG2D are generally not expressed on healthy cells but are induced on the surface of malignant cells. In humans, NKG2D is engaged by several ligands, namely MHC class I polypeptide-related sequence A and B (MICA and MICB) and six members of the UL16-binding glycoproteins 1–6 (ULBP1-6) [3]. However, tumor cells develop mechanisms to escape innate immune surveillance [4]. These strategies include the proteolytic shedding and release of soluble NKG2D-L to render target cells invisible to an NKG2D-dependent NK cell attack. Soluble ligands for NKG2D do not only passively block receptor activation, but moreover, cause the downregulation of receptor surface expression for immune evasion [5]. Many studies validated the critical role of the NKG2D/NKG2D-L system for tumor development in cancer patients and tumor mouse models [6, 7]. This is highlighted by a recent study, showing that inhibition of MICA/MICB shedding and thus release of soluble ligands from the cell surface, prevented tumor growth in immunocompetent mouse models and reduced melanoma metastasis in a humanized model [8]. Reactivation of anti-tumor immunity via targeting of the NKG2D/NKG2D-L axis is therefore a promising therapeutic approach.

It has been previously described that the transcriptional upregulation of NKG2D-L mainly depends on the activation of cellular stress signals, although the specific molecular mechanisms remain largely unknown. In general, inducible expression of NKG2D-L is observed in response to oncogenic transformation, cell cycle alterations, and DNA damage [1]. NKG2D-L induction in mice was attributed to a hyperproliferative state which activates the E2F transcription factor and p53, allowing the transcription of NKG2D-L. Furthermore, indirect effects via p53-mediated cytokine release were reported. In addition, NKG2D-L expression is regulated post-transcriptionally. DNA damage-associated factors such as TBK1 and IRF3 stabilize NKG2D-L mRNA [9]. The expression of NKG2D-L is further regulated by microRNAs repressing the translation of NKG2D-L including MICA [10, 11]. Post-translational regulation of the NKG2D-L surface expression via protein modifications has also been reported, and a shedding-induced release of ligands either in soluble form or in association with extracellular vesicles [1]. Recently it was shown that neddylation through the enzyme NEDD8 decreases MICA expression in multiple myeloma, suggesting that neddylation inhibitors may provide novel therapeutic options [12].

The absence of NKG2D-L expression on AML cells in humans and mice was recently attributed to transcriptional repression mediated by the poly (ADP-ribose) polymerase and transcription factor PARP1 [13]. Of note, the PARP1-dependent absence of NKG2D-L on acute leukemia cells correlated with cancer stem cell properties, supporting immune evasion of these stem cells and finally leading to tumor development. Inhibiting PARP1 to allow expression of NKG2D-L is therefore a promising therapeutic approach targeting therapy-resistant and tumor-forming leukemia stem cells. However, the clinical application seems to be limited due to side effects [14]. Transcription factors, especially those which can be targeted with small molecules to induce NKG2D-L expression remain to be identified.

AML is a genetically heterogeneous cancer of hematopoietic stem and progenitor cells. The overall 5-year survival rate particularly in elderly patients is less than 10%. Most patients achieve complete remission by standard chemotherapy, but later relapse is a major concern. Allogeneic hematopoietic stem cell transplantation is a curative treatment option but not eligible for all patients. As stem cells play a pivotal role in relapse, novel treatment options, *e.g.* via upregulation of NKG2D-L or targeting the NKG2D/NKG2D-L axis are holding promise [15].

Therefore, we aimed to identify specific transcription factors, which regulate the inducible expression of the NKG2D-L MICA. Previously we showed that the major acetyltransferases CBP and p300 have a robust, mandatory, and general impact on the upregulation of mouse and human NKG2D-L in response to histone deacetylase inhibition (HDACi) [16]. However, the responsible transcription factors remain unknown and therefore we used the HDACi-dependent MICA expression as a model to identify such factors.

A better understanding of the transcription factors controlling the NKG2D-L expression will contribute to the development of novel therapeutic strategies aiming at an increased NKG2D-L expression on the AML cell surface and thus potential killing by NK cells.

## Methods

### Cell lines

The human embryonic kidney 293 cell line (HEK293, DMSZ ACC305) and the acute myeloid leukemia cell lines HL60 (DMSZ ACC3), NB4 (DMSZ ACC207), and MonoMac6 (MM6, DMSZ ACC124) were cultivated as recommended. NB4 and MM6 cells were kindly provided by H. Daniel Lacorazza, Houston, USA.

### Inhibitors and chemotherapeutic agents

Cells were treated with LBH589 (SEL-S1030, Selleck Chemicals), kenpaullone (S7917, Selleck Chemicals), araC (cytarabine in 0.9% NaCl solution) and different concentrations of APTO253 (S6963, Selleck Chemicals). LBH589 and APTO253 were dissolved in dimethyl sulfoxide (DMSO).

### RNA isolation, cDNA synthesis, and RT-qPCR

Total RNA was extracted using the NucleoSpin RNA Kit (Macherey–Nagel). 500 ng RNA were used for cDNA synthesis using the RevertAid RT Reverse Transcription Kit (Thermo Scientific Fisher) according to the manufacturer's instructions. The qPCR reaction was carried out using the ABsolute qPCR SYBR Green Mix (Thermo Fisher Scientific) and a Thermo Cycler Mx3005P (Stratagene). The level of target gene expression was measured using the △△Ct method and normalized to *RPL27* gene expression. Each gene was tested in technical triplicates and all experiments were performed in at least three independent experiments.

The following primers were used:

*MICA* forward (fw.) 5’-CTGCAGGAACTACGGCGATATCT-3’ and reverse (rev.) 5’-CCCTCTGAGGCCTCGCTG-3’; *MICB* fw. 5’-AGAAGAAAACATCAGCGGCAG-3’ and rev. 5’-CATCCCTGTGGTCTCCTGTC-3’; *RPL27* fw. 5’-AAAGCTGTCATCGTGAAGAAC-3’ and rev. 5’-GCTGTCACTTTGCGGGGGTAG-3’; Upstream promoter *MICA* fw. 5’-GGCGCCTAAAGTCTGAGAGA-3’ and rev.´5 CAGCAAGAAACCCTGACTGC-3’; Standard promoter *MICA* fw. 5’-GGCTGGCATCTTCCCTTTTG-3’ and rev. 5’-CAGCAAGAAACCCTGACTGC -3’; *HBG* fw. 5’-GCAAAGAGAGTGAGGGTCGG-3’ and rev. 5’-TGGATGATCTCAAGGGCAC-3’**;** *KLF4* fw. 5’-GAAATTCGCCCGCTCCGATGA-3’ and rev. 5’-CTGTGTGTTTGCGGTAGTGCC-3’; *c-MYC* fw. 5’-CCTCCACTCGGAAGGACTATC-3’ and rev. 5’-TGTTCGCCTCTTGACATTCTC-3’.

### Transcription activation based on CRISPR/dCas9 activation system

2.5 × 10^5^/ mL HEK293 cells were seeded in 6 well-plate. Cells were transiently co-transfected with 2.5 µg of SP-dCas9-VPR activation expression vector (Plasmid # 63798, Addgene) and 2.5 µg gRNA vector (Plasmid # T141819, Addgene) targeting the locus of interest using 5 µl of lipofectamine 2000 (Thermo Fisher). 48 h post-transfection, cells were harvested, RNA was isolated and subjected to cDNA synthesis followed by qPCR analysis.

### enChIP-qPCR

5 × 10^6^ / 20 mL of HEK293 cells were seeded in a 15 cm culture dish for 20 h. Cells were transfected with 8 µg of pEF1a-FB-dCas9-puro expression vector (Plasmid # 100547, Addgene) and 8 µg of gRNA vector targeting the locus of interest with 40 µL lipofectamine 2000. 48 h post-transfection cells were treated with 100 nM LBH for 4 h followed by Chromatin immunoprecipitation (ChIP). For precipitation, 4 µg Cas9 (Sigma Aldrich) or 4 µg IgG (Sigma Aldrich) per immunoprecipitation (IP) were used. DNA was purified using a PCR purification kit (Qiagen) according to the manufacturer’s instructions. For the PCR, the following primers were used: *MICA* fw. 5’-CGTGCTTATGAAGTTGGA-3’ and rev. 5’-AGACCTGGGGAGATTTAG-3’; *PDK4* fw. 5’-GTATGTGTACTGGGGGGAC-3’ and rev. 5’-CAGATGGCTCTTTTCGTTCC-3’.

### enChIP-mass spectrometry (MS)

For the enChIP-MS analysis, the enChIP procedure was performed as described above, using 2–3 × 10^8^ cells per condition. For chromatin pre-clearing, 2 ml of a blocked mixture of protein A and protein G Dynabeads was used. 120 μg of Cas9 (Thermo-Fisher) antibody coupled with 1500 µL of Protein A + G Dynabeads were added for precipitation. Samples were stored with cocktail protease inhibitors at -80 °C until they were proceeded by off-bead digest and high pH reversed-phase separation, followed by LC-MS2 analysis utilizing label-free quantitation.

### Dual-luciferase reporter assay

The human promoter region of *MICA* (583 bp within the standard promoter region) was cloned into a pGL3-Basic luciferase reporter vector via double-digestion with *Kpn*I and *Hind*III. HEK293 cells were co-transfected with a 2.5 µg pGL3-MICA-luciferase reporter vector and 200 ng of the expression vector, together with 100 ng pRL-TK-renilla vector (Promega). 48 h post-transfection cells were lysed and luciferase activity was measured using the Dual-luciferase reporter system (Promega, Madison, WI, USA) according to the manufacturer's instructions. Relative luciferase units (RLU) were calculated by measuring the firefly and renilla luciferase ratio and compared to the appropriate controls. The plasmids pcDNA3-Flag-hKLF4; pLVX-hKLF4-TetOne-puro (iKLF4) and pLVX-hKLF4-delC-TetOne-puro (iDN-KLF4) were kindly provided by Takashi K. Satoh and Lars French, Munich, Germany.

### Flow cytometry

7 × 10^5^ HL60 cells were seeded in 6 well plates and treated with LBH589, APTO253, or DMSO for 18 h. Cells were resuspended in 600 µL buffer (1 × PBS containing 1% FSC). 100 µL of the sample were used and blocked with 50 µL (diluted 1:100 in staining buffer) of mouse γ-globulin (015–000-002, Jackson Immuno Research) per sample for 15 min at 4 °C. Cells were incubated with 1 µL MICA/B (320914, AF 647 Biolegend) or IgG2a1 (400234, AF 647 Biolegend) antibody for 30 min at 4 °C in the dark. Cells were washed with staining buffer and 1 µl 50 µg/ml propidium iodide (PI, Sigma Aldrich) was added per sample (Sigma Aldrich). Cell death and MICA/B expression were measured by flow cytometry using a FACS Canto II cytometer (BD Bioscience) and analyzed by FACS Diva software.

### Killing assay

Peripheral blood mononuclear cells (PBMCs) were purified by ficoll density centrifugation using LSM1077 (Sigma-Aldrich). NK cells were isolated using a negative selection NK cell isolation kit (Miltenyi Biotec, Bergisch Gladbach, Germany). Isolated NK cells were cultured in IMDM medium (Gibco^TM^) with 10% FCS, 1% P/S, and 10 U/mL IL-2 and rested at 37 °C overnight. For killing assays, HL60 cells were stained with 5 µM CellTracker^TM^ Violet BMQC (Life Technologies) fluorescent dye in serum-free medium at 37 °C for 45 min. Then, FCS was added to the cells at a final concentration of 20%. Cells were resuspended in fresh medium and seeded accordingly in IMDM medium with 10% FCS and 1% P/S in U-shaped 96-well plates. NK cells were added to the target cells in ratios ranging from 1.25:1 to 10:1. The cells were co-cultured for 3 h before they were harvested and centrifuged at 300 × g for 5 min. The supernatant was discarded, and the cells were resuspended in 200 µL PBS before they were stained with 1 µl 50 µg/ml PI per sample. Cell death was measured by flow cytometry using a FACS Canto II cytometer (BD Bioscience) and analyzed by FACS Diva software.

### ELISA

IFN-γ and TNF-α in the supernatant were quantified using the DuoSet™ ELISA from R&D Systems (DY285B-05) and (DY210-05), respectively, according to the manufacturer's instructions.

### siRNA knockdown

For KLF4 knockdown experiments, 3 × 10^5^ HEK293 cells were seeded in 6-well plates. KLF4 knockdown was performed using lipofectamine 2000 and 50 nM siKLF (sc-35480, a pool of 3 target-specific 19–25 nt siRNAs, Santa Cruz Biotechnology, Inc). A non-targeting siRNA Pool#1 (D-001206–13-05, Dharmacon) was used as a control. 24 h later, cells were treated with 100 nM LBH589 or DMSO for 4 h before cells were collected for qRT-PCR.

### Statistics

GraphPad Prism was used for depicting bar charts of means with standard error of the mean (SEM) as indicated. GraphPad Prism was used for the calculation of significances (*p < 0.05, **p < 0.01, ***p < 0.001, ****p < 0.0001) for the statistical test applied as indicated.

## Results

### Targeting the *MICA* standard promoter using the enCHiP method for subsequent single-locus proteomics

**Fig. 1:**
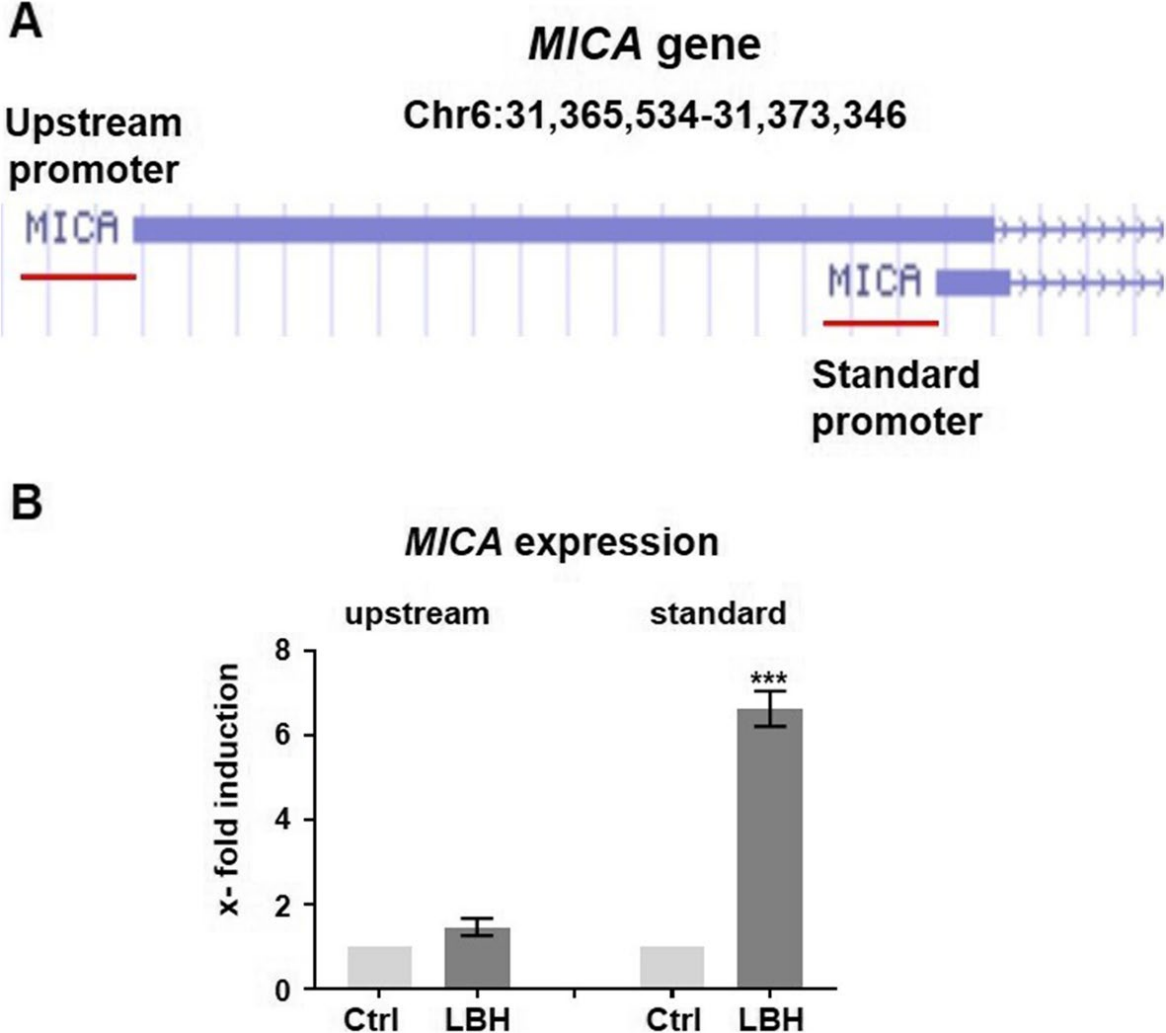
LBH589 regulates the transcription of *MICA* from the standard promoter but not from the upstream promoter. **A** Section from the UCSC Genome Browser (Human, GRCh37/hg19) to depict the *MICA* upstream and standard promoter regions (red lines) and the corresponding exon 1 (blue boxes). The standard promoter loci, which are targeted by the guide RNAs are provided in the methods section. **B** RT-qPCR analysis to measure the expression level of the upstream transcript and the standard transcript in HEK293 cells treated with 100 nM LBH589 for 4 h. Data represent the mean ± SEM of three independent experiments. Significances were calculated using an unpaired student's t-test

Recently it was described that *MICA* transcription is controlled by both an upstream and a standard promoter with transcripts that differ in exon 1 [10] (Fig. 1A). To identify the respective promoter regions responsible for inducible and HDACi-regulated *MICA* expression, we treated HEK293 cells with the HDACi LBH589. Subsequently, RT-qPCR was performed to quantitatively assess the expression of transcripts that are under the control of the two promoters. The results clearly indicated, that HDACi treatment predominantly regulated the standard promoter-dependent *MICA* transcription (Fig. 1B).

**Fig. 2:**
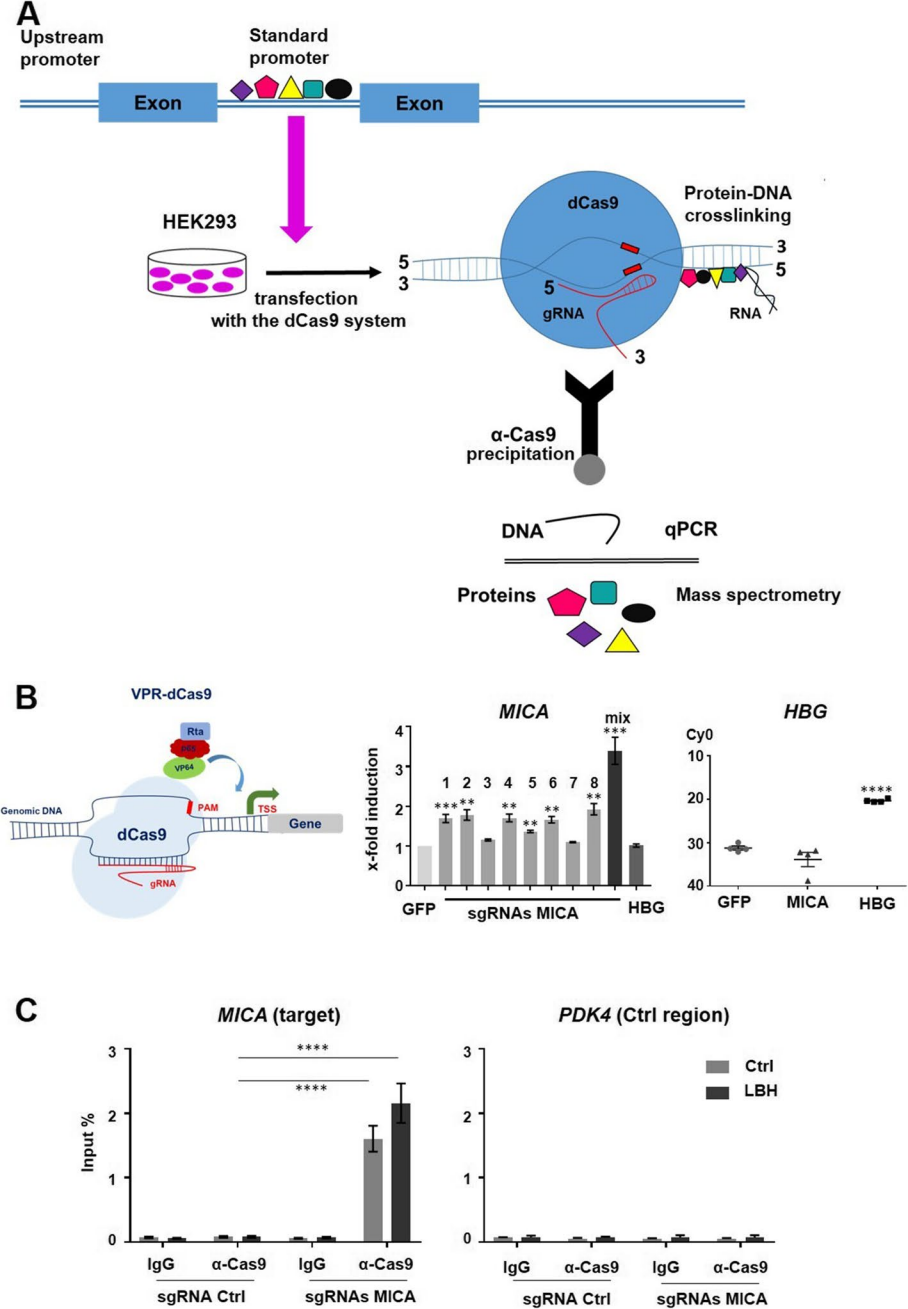
Establishment of the enChIP method. **A** Graphical summary of the enChIP approach. **B** Induction of *MICA* gene expression through the SP-dCas9-VPR/gRNA activation system. RT-qPCR was performed 48 h after transient transfection of HEK293 cells with dCas9/VPR (Rta, p65, VP64) and sgRNAs 1–8 targeting the *MICA* promoter locus, a mix of sgRNAs 1,2,4,6, and 8, as well as a *HBG* promoter locus-targeting sgRNA as a positive control and a GFP-targeting one as a negative control. PAM: protospacer adjacent motif, TSS: transcription start site. The y-axis for *HBG* depicts Cy0 for the cycle threshold (CT) at which a measurement signal was present during cyclic amplification. **C** enChIP using the pEF1a-FB-dCas9/gRNA system to isolate the *MICA* promoter genomic region. Transient transfections of HEK293 cells with pEF1a-FB-dCas9 and a mix of sgRNAs to target the *MICA* promoter locus, as well as *GFP-*targeting control sgRNA were performed. Transfected cells were subjected to enChIP and the target locus was isolated using dCas9-targeting immunoprecipitation. RT-qPCR analysis was performed to validate an enrichment of the *MICA* promoter, for which the *PDK4* region was used as a negative control. Data (**B**, **C**) represent the mean (±SEM) of three or four biological replicates. Statistical analysis was performed with an unpaired student's t-test

To analyze the locus-specific chromatin-regulating proteome, we modified a technique initially described as in situ capture of chromatin interactions by an inactive Cas9 (dCas9) [17] (see Fig. 2A for an overview of the SP-dCas9-VPR/gRNA activation system). HEK293 cells were used as a model because our previous experiments showed that the CBP/p300-dependent upregulation of NKG2D-L could be recapitulated in these cells [16]. In brief, a set of eight single guide RNAs (sgRNAs) targeting the *MICA* standard promoter region (from -85 to -413 bp) was individually co-transfected with a vector expressing dCas9 fused to the general transcription factor activation domains of VP64, p65, and Rta (SP-dCas9-VPR) known to activate gene expression upon targeting the DNA [18] (Fig. 2B). Promoter binding of the sgRNAs-loaded SP-dCas9-VPR was expected to induce *MICA* transcription. Indeed, RT-qPCR validated five of the eight sgRNA candidates to significantly increase *MICA* transcription, with a mixture of them resulting in optimal induction efficiency (Fig. 2B). A control sgRNA targeting a GFP sequence did not impact *MICA* expression. As a positive control, we used the *HBG* promoter locus, which was induced by a specific sgRNA but is not affected by *GFP*- or *MICA*-targeting sgRNAs (Fig. 2B, right panel). Thus, we confirmed that the designed sgRNAs specifically targeted and significantly affected the *MICA* promoter. In the following, we used these sgRNAs in combination with dCas9 to isolate the *MICA* promoter and its associated proteins using a Cas9 antibody.

Given the expectation of low abundance for sequence-specific binding factors, the study aimed to identify, we upscaled the approach (2–3 × 10^8^ cells starting material). A robust and significant precipitation of the *MICA* locus was observed using non-treated cells and upon treatment with LBH589, which was used to induce *MICA* expression (Fig. 2C, left panel). Confirming the specificity of the method, no precipitation of the control region *PDK4* was observed (Fig. 2C, right panel) and the CRISPR/dCas9 system did not interfere with the LBH-mediated MICA induction (Supplement Figure S1A). As a control, a guide RNA targeting *GFP* was used (sgRNA Ctrl).

**Fig. 3:**
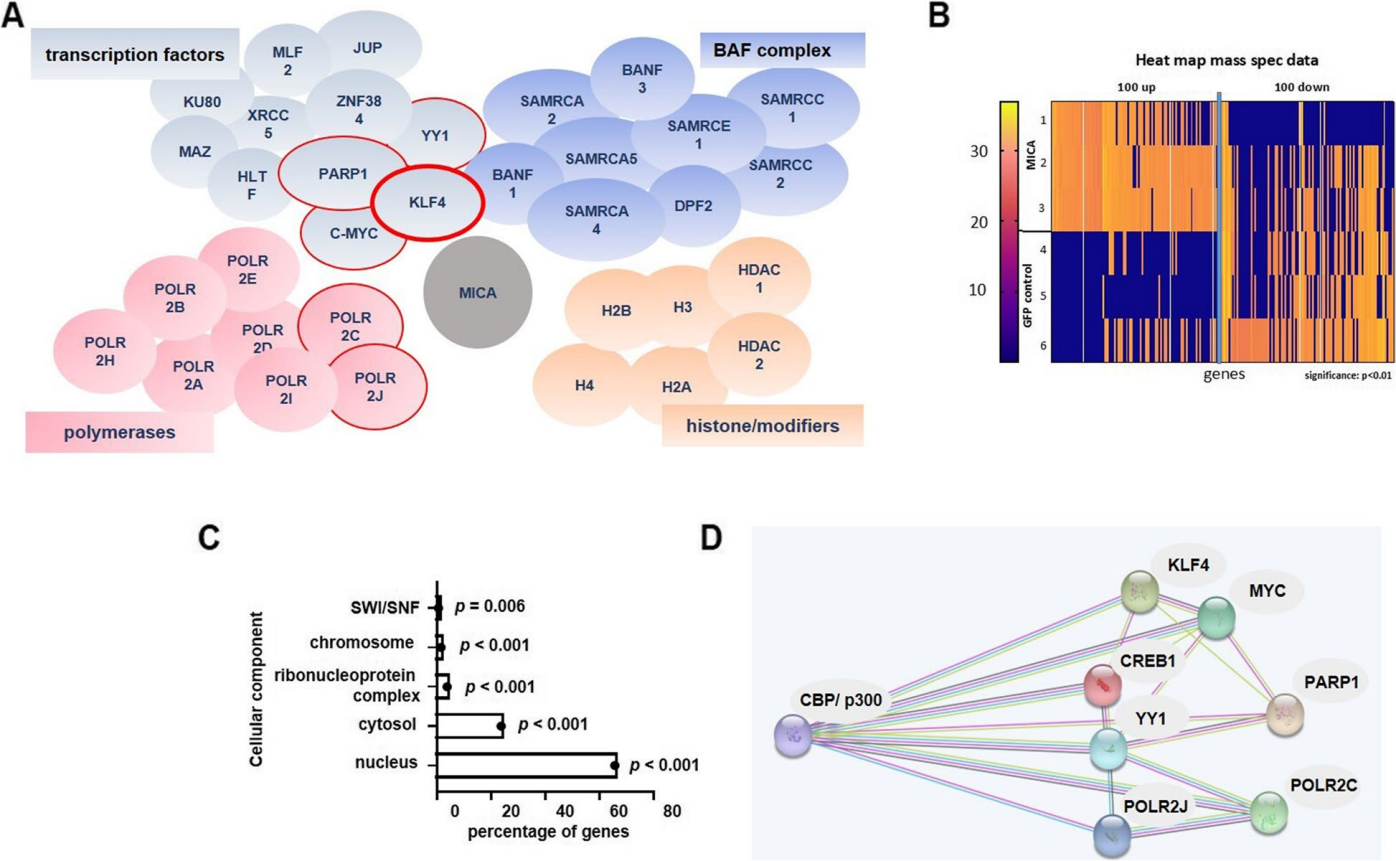
EnChiP-MS targeting the MICA locus. **A** Candidate proteins, putatively involved in *MICA* regulation (full list see Supplement Table S1). Factors with red edges are already identified regulators and transcription factors with binding motifs in the *MICA *promoter or/and factors regulated by CBP/p300. **B** Heat map of label-free quantitation of MS data of three biological replicates for the MICA (1,2,3) and the GFP control immunoprecipitations (4,5,6). Note the divergence of samples 1 and 6, reflecting experimental variation and/or the highly dynamic nature of chromatin. **C** Enrichment analysis to catalog proteins according to their cellular compartments using a functional enrichment analysis tool (http://www.funrich.org). About 50% of the proteins annotated to cytosol were also detectable in the nucleus and of these about 40% were annotated to the nucleolus. **D** Protein–protein interaction network for candidate transcription factors constructed using the STRING database (https://string-db.org). Associations are specific and meaningful but do not necessarily imply physical interaction. Interactions are indicated as experimentally determined (pink); from curated databases (blue); predicted from gene neighborhood (green), gene co-occurrence (dark blue), or protein homology (purple)

The subsequent mass spectrometry-based proteomic analysis of the immunoprecipitated protein mixture (LC-MS2 with label-free quantitation) identified approximately 1500 proteins in the MICA samples (*n* = 3). Among them were transcription factors, components of the chromatin remodeling complex such as BAF, polymerases, and histone modifiers as expected (Fig. 3A). The high number of total proteins, however, suggests many of them represent background, such as highly abundant proteins. Nevertheless, heat maps comparing immunoprecipitates targeting MICA or GFP and showing the 100 most enriched/decreased proteins in the MICA over GFP samples reveal differences between them (Fig. 3B), although variability between the replicates is seen (*n* = 3). In general, an enrichment of cellular components including nuclear and chromatin factors was observed (Fig. 3C). To screen for candidate transcription factors regulating *MICA*, we focused on candidates enriched in MICA samples, generally involved in gene regulation, and to be targeted by CBP/p300 (Fig. 3D).

**Fig. 4:**
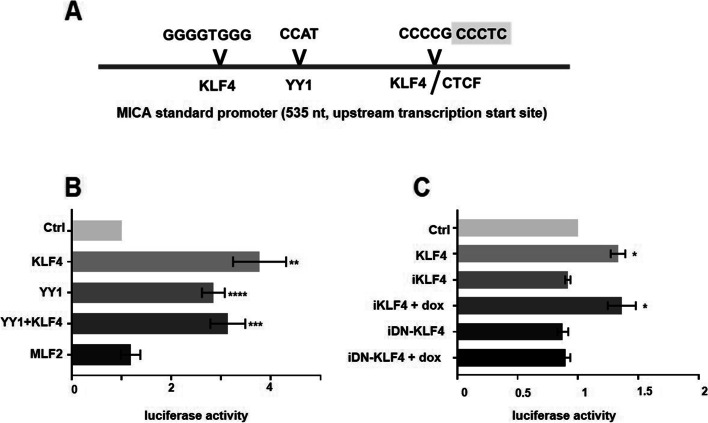
KLF4 and YY1 regulate *MICA* expression. **A** A 535 bp *MICA* promoter construct with binding sites for YY1, KLF4, and CTCF was used for the reporter assays. **B** Dual-Luciferase® Reporter (DLR™) Assay to determine the impact of KLF4, YY1, and MLF2 on the *MICA* standard promoter. Equal amounts of expression vector DNA were transfected. As a control (Ctrl) an expression vector encoding GFP was used. The luciferase activity was determined according to firefly / renilla normalized to the GFP control. **C** HEK293 cells were transiently transfected with the KLF4 expression plasmid as a positive control, an inducible KLF4 (iKLF4) or an inducible mutant lacking the DNA-binding domain (iDN-KLF4). The expression was induced by doxycycline (dox) using the Tet-on system. Activation of the *MICA* reporter was measured (see **B**). Data are shown as the mean (± SEM) of three independent experiments. Significance was calculated using an unpaired t-test. See supplemental figure S2  for validation of the inducible KLF4 expression by Western blot

Using JASPAR (https://jaspar.uio.no), an open-access transcription factor binding profile database, we identified binding motifs for two of the transcription factors detected by MS, namely KLF4 and Yin Yang 1 (YY1) (Fig. 4A). The KLF4 binding site partly overlaps with a binding motif for the chromatin-organizing factor CTCF, which promotes the formation of chromatin loops for activation of transcription [19]. KLF4 transcriptional activity is regulated by the p300/CBP coactivator family [20], and both YY1 as well as KLF4 directly bind to CREB-binding proteins [21] known to be involved in *MICA* regulation [16]. In addition, KLF4 is essential for the cellular response to γ-irradiation-induced DNA damage [22], a process also known to induce NKG2D-L expression in mice [23]. Furthermore, some of the identified transcription factors, including KLF4 and YY1, bind directly to the *MICA* standard promoter region in proximity to the CpG island (ChIP data, ENCODE database, see Supplement Figure S1B). Taking together, based on the enChIP results, we set out to analyze the role of the identified transcription factors with a focus on KLF4 in more detail.

### KLF4 regulates reporter gene expression regulated by the *MICA* promoter

In a first step, we analyzed a *MICA* promoter construct with transcription factor binding sites for KLF4 and YY1 in a dual-luciferase reporter assay (Fig. 4A), to explore whether KLF4, YY1, or the transcription factor MLF2 (with an unknown DNA-binding motif) regulate the reporter gene expression. Transfection of KLF4, YY1, or their combination all induced the expression of the *MICA* reporter significantly, whereas MLF2 expression did not show any effect (Fig. 4B). In addition, we tested the activity of a doxycycline-inducible KLF4 expression vector and a KLF4-deletion mutant lacking the DNA-binding domain (DN-KLF4) (for protein expression see Supplement Figure S2). In the absence of doxycycline, none of the constructs showed any activation as expected. Upon doxycycline induction, only KLF4-WT but not the deletion mutant (DN-KLF4) was capable of activating the *MICA* promoter (Fig. 4C) suggesting that direct binding to the promoter was involved, although indirect effects could not be excluded. These data indicate that KLF4 acts as a positive regulator of *MICA* expression.

**Fig. 5:**
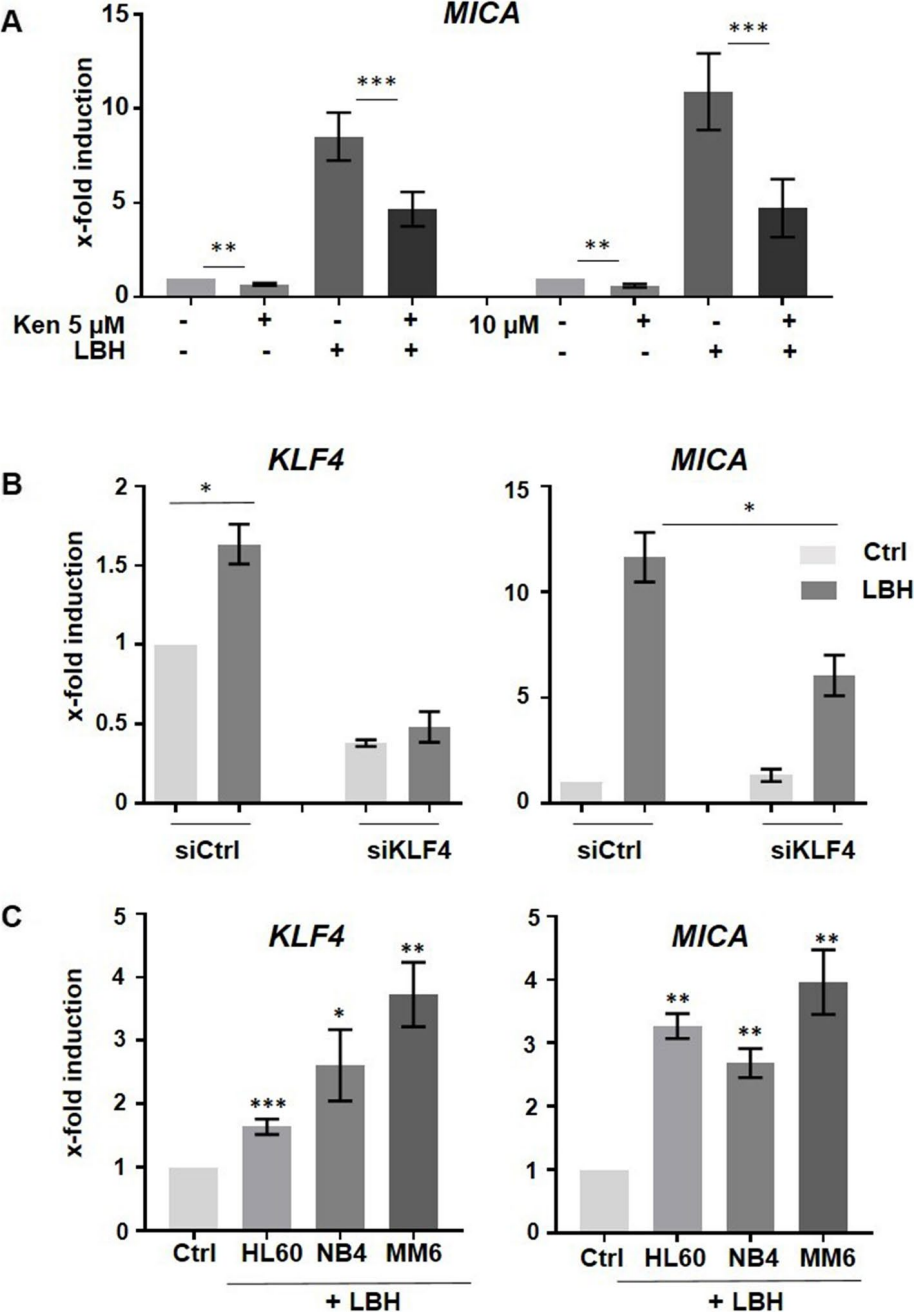
LBH589-mediated *MICA* upregulation is diminished in KLF4-depleted cells. **A** Cells were treated with 5 µM or 10 µM KLF4 inhibitor kenpaullone (ken) for 4 h prior to LBH589 treatment and subjected to RT-qPCR to measure *MICA* mRNA. See supplemental Western Blot Figure S3 for KLF4 protein expression. **B** HEK293 cells were treated with siRNA (control and *KLF4*) for 24 h followed by 100 nM LBH589 4 h treatment. The expression of the *KLF4* and *MICA* genes was measured by RT-qPCR analysis. Data represent the mean (± SEM) of two biological experiments each measured in duplicates. Significance was calculated using a Mann–Whitney test. **C** HL60, NB4, and MM6 AML cell lines were treated with 100 nM LBH589 for 4 h and RT-qPCR was performed to detect mRNA expression levels of *MICA* and *KLF4*, respectively. The mean (± SEM) of three or four independent experiments is indicated and significance was calculated using an unpaired student’s t-test

These results prompted us to investigate whether KLF4 is critically involved in the upregulation of the *MICA* gene in response to LBH589 by pharmacological- and siRNA-mediated approaches. Therefore, HEK293 cells were treated with KFL4 inhibitor kenpaullone (ken) prior to LBH589 treatment. The decrease in KLF4 protein expression was associated with a significantly impaired *MICA* upregulation in response to LBH589 (Fig. 5A).

Similar results were observed when KLF4 was knocked down by siRNA, as *MICA* expression was significantly diminished in siKLF4-transfected cells as compared to control siRNA cells (Fig. 5B), indicating that KLF4 is necessary for *MICA* regulation by LBH589 stimulation.

### HDACi treatment of AML cells induces both, *KLF4* and *MICA* expression

To evaluate whether, reciprocally, HDACi-mediated upregulation of MICA in AML cells is linked with an elevated expression of KLF4, we treated three human AML lines with LBH589 and subsequently examined the expression levels of *MICA* and *KLF4* by RT-qPCR. LBH589 treatment resulted in an upregulated *MICA* expression in all cell lines tested. This *MICA* regulation was correlated with a significant increase in *KLF4* expression, which is in line with its role in the positive regulation of *MICA* (Fig. 5C).

**Fig. 6:**
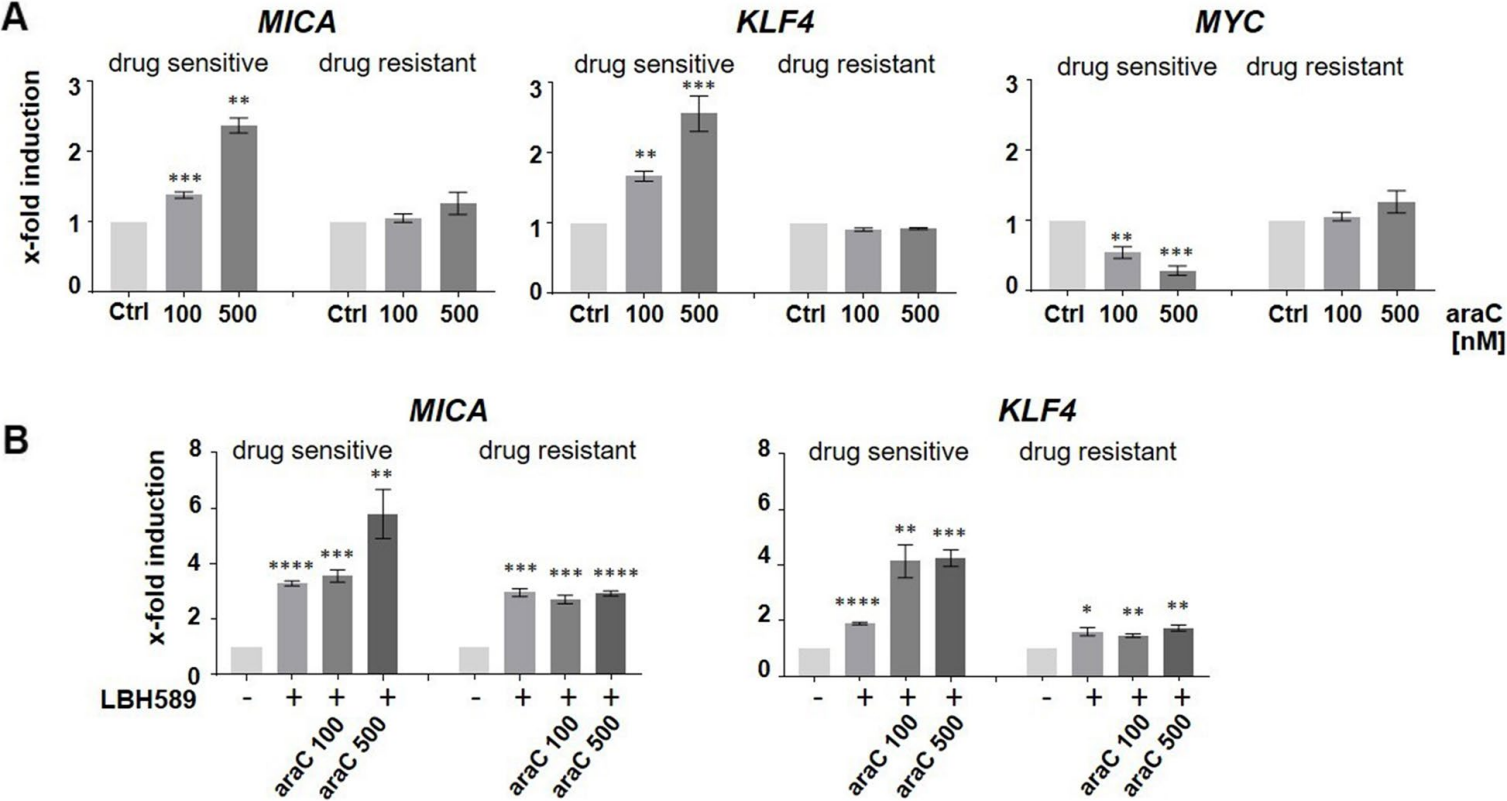
*KLF4* and *MICA* expression in response to araC in cytarabine-resistant and sensitive HL60 cells. **A** AraC sensitive and resistant HL60 were treated with araC for 24 h prior to RT-qPCR analysis for *MICA*, *KLF4,* and *MYC*. **B** HL60 cells were treated with 100 and 500 nM araC for 24 h, followed by 100 nM LBH589 for 4 h. RT-qPCR analysis was performed, and the expression level of *KLF4* and *MICA* was measured. Data represent the mean (± SEM) of three biological replicates. Significance was calculated with an unpaired student’s t-test

Besides HDACi, DNA damage, *e.g.* caused by chemotherapeutics, also is known to result in an upregulation of *MICA*. To explore the role of KLF4 in DNA damage-mediated *MICA* induction, we analyzed the expression of *KLF4* and *MICA* in the AML cell line HL60 in response to cytarabine (araC), a drug known to induce DNA damage in AML [24]. As expected, araC induced *MICA* expression in HL60 cells, which correlated with an enhanced expression of *KLF4* and inversely with a decrease in *MYC* expression (Fig. 6A). These effects on *MICA*, *KLF4,* and *MYC* expression were notably abolished entirely in araC-resistant HL60 cells. We further asked whether the LBH589-mediated *MICA* upregulation was diminished in chemotherapy-resistant cells as well. Treatment with LBH589 as a single agent or in combination with araC induced the expression of *MICA* (and *KLF4*) in both resistant as well as sensitive cells (Fig. 6B). However, an additive effect of LBH589 and araC was only observed in sensitive cells. We conclude that *KLF4* is involved in both, HDACi and DNA damage-mediated *MICA* regulation and that DNA damage-induced upregulation is abolished in chemoresistant cells, which fail to express *KLF4*.

### APTO253 induces *MICA* expression in AML cells

Next, the regulation of *MICA* in response to the small compound APTO253, known to induce KLF4 expression, was analyzed in AML cells. APTO253, originally described as a MYC inhibitor is a drug developed for the clinical treatment of hematological malignancies [25, 26].

**Fig. 7:**
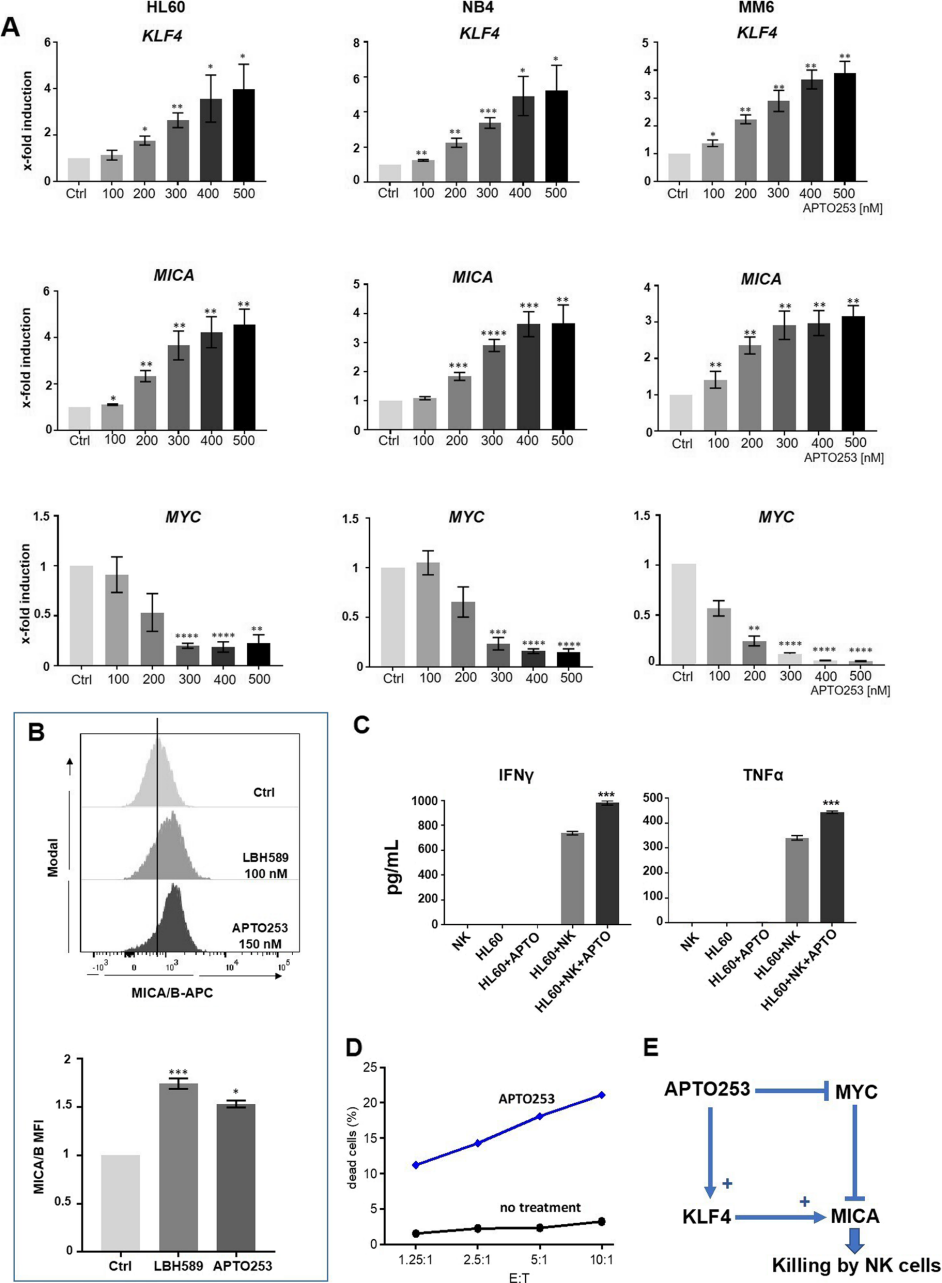
APTO253 treatment induces expression of *KLF4* and *MICA* but represses *MYC* in AML cell lines. **A** HL60, NB4, and MM6 cell lines were incubated with APTO253 at concentrations indicated for 18 h followed by RT-qPCR to measure *KLF4*, *MICA,* and *MYC* expression. Data are shown as the mean (± SEM) of three independent experiments. **B** Expression level of surface MICA on untreated, LBH589- or APTO253-treated HL60 cells measured by flow cytometry. A histogram of one experiment and the mean (± SEM) of three biological replicates is depicted. Significance was calculated using an unpaired t-test (**A**,**B**). **C** HL60 cells, either treated with APTO253 or solvent were incubated with NK cells in a ratio of 1:10 as described in **C**) and the supernatant of mono- and co-cultures was used to measure released IFNγ and TNFα by ELISA. *n* = three technical replicates. **D** No treatment (control vehicle) and APTO253-treated HL60 cells were used as target cells in an NK cell-dependent killing assay using NK cells isolated from healthy donors (one out of three representative experiments). The ratio of target cells to effector NK cells is indicated (E:T). The basal cell death of HL60 ± APTO253 without any NK cells was measured in parallel to the co-cultured HL60. These values ranged from ~ 2–10% and were then subtracted from the co-culture values. **E** Graphical summary

APTO253 treatment induced a robust expression of both, *KLF4* and *MICA*, in a dose-dependent manner in AML cells (Fig. 7A). We observed a strong correlation between the extent of upregulation for *KLF4* and *MICA*, which inversely correlated with the expression of *MYC* (Fig. 7A). In line with these findings, the HDACi-mediated up-regulation of *MICA* is also associated with MYC suppression in AML cell lines (Supplement Figure S4). Interestingly, the response to APTO253 was restricted to tumor cells, whereas lymphocytes from healthy donors did not up-regulate *MICA* or *KLF4* (Supplement Figure S5).

To address the functional relevance of MICA induction in response to APTO253, the protein surface expression of MICA was measured by flow cytometry. APTO253-treated HL60 cells revealed a higher NKG2D-L expression (Fig. 7B). In line, NK cells isolated from healthy donors released higher amounts of the pro-inflammatory cytokines IFNγ and TNFα upon co-culture with APTO253-treated HL60 cells in comparison to untreated HL60 cells (Fig. 7C). Moreover, treated cells were much better recognized and killed by NK cells (Fig. 7D). This unraveled a link between APTO253 treatment and innate immune cell activation. We concluded that APTO253 treatment, which affected the expression level of MYC, KLF4, and MICA resulted in better NK cell recognition of AML cells (Summary Fig. 7E).

## Discussion

The anti-tumor effect of NK cells in leukemia was reported in several pre-clinical and clinical studies. Their therapeutic success is, however, limited due to immune evasion strategies of the leukemic blasts [27, 28]. Approaches to enhance the susceptibility of leukemic blasts to NK cell-dependent killing, therefore, appear promising.

The primary objective of this study was to identify pharmacologically accessible transcription factors, which induce MICA expression in AML cells by using a novel engineered ChIP approach in combination with mass spectrometry. Among the locus-associated chromatin components, we identified KLF4 and provide evidence for its critical role in the transcriptional regulation of *MICA*. Our conclusions are based on the findings that 1) the *MICA* promoter harbors KLF4 binding motifs and is regulated by KLF4 and that 2) the established HDACi-mediated upregulation of MICA is diminished upon chemical inhibition or genetic ablation of KLF4. Of note, KLF4 activity is known to be regulated by CBP/p300 acetyltransferases, which are the critical enzymes in the HDACi-mediated upregulation of *MICA* [16]. Moreover, we demonstrate that 3) the small molecule APTO253, a KLF4 inducer, leads to the upregulation of *MICA* in AML cells. In line with this observation, 4) upregulation of *MICA* in response to DNA damage is also associated with KLF4 induction and abolished in chemoresistant cells, which fail to express KLF4. Interestingly and pointing towards the clinical relevance of these data, 5) APTO253-treated AML cells express surface MICA and are more susceptible to NK cell-mediated killing, although we cannot exclude that other receptor-ligand interactions may contribute to increased killing.

The identification of the transcription factor network responsible for the inducible expression of a target gene remains difficult, as the chromatin composition is complex and dynamic, and single-locus proteomics remains technically challenging [29, 30]. To filter promising candidates, we considered transcription factors fulfilling the following criteria as putative *MICA* regulators: first, regulation by CBP/p300-mediated acetylation and, second, binding motifs (if known) within the *MICA* promoter. Besides KLF4 we also detected PARP1, which was recently described to inhibit MICA expression on AML cells as part of the tumor immune evasion strategy [13] which validates the approach. Other factors passing the selection criteria, including the YY1 protein, were so far not described in the context of *MICA* transcriptional regulation. YY1 is known to antagonize PARP1-mediated inhibition of expression of a chemokine ligand by enhancing its transcription [31] and thus may play an analogous role in PARP1-mediated inhibition of NKG2D-L expression.

Among the candidates, we focused on KLF4, because KLF4 can be induced by APTO253, a small molecule that is already in clinical testing. KLF4 is a member of the family of SP1-like transcription factors and is involved in cellular processes such as proliferation, differentiation, apoptosis, and somatic cell reprogramming [32]. As YY1, KLF4 activates gene expression after interaction with the p300/CBP coactivator family [20], and YY1 and KLF4, both, bind directly to CREB-binding protein [21]. KLF4 has been shown to function as a tumor suppressor or oncogene in cell-dependent contexts [33]. Although the role of KLF4 in AML is controversially discussed, it has been shown to play an oncogenic role in AML cell lines and the deletion of *KLF4* by CRISPR/Cas9 suppresses cell growth and induces apoptosis [34]. These experiments were, however, performed in immunodeficient mouse models, and a putative innate immune response to NKG2D-L positive AML was not addressed. KLF4 is one of the Yamanaka factors able to induce pluripotent stem cells and was recently shown to promote leukemia stem cell division and stemness [35]. This finding may seem paradoxical but makes sense considering that the NKG2D-L expression is an early cellular response to malignant transformation in order to alert the innate immune system to dangerous cells – which is characterized by high KLF4 expression. In line with that, RNA expression data of sorted NKG2D-L negative AML patient cells (with stem cell properties) revealed a significantly lower KLF4 expression as compared to NKG2D-L positive AML patient cells (no stem cell properties) (*p* = 4.4 × 10^–44^, see supplement in [13]). Other studies showed KLF4 expression is downregulated in AML patients [36, 37].

Novel approaches to treat AML patients include HDACi treatment, but therapy responses appear modest so far and are associated with side effects [38]. Recently promising anti-tumor activity was demonstrated in pre-clinical experiments when HDACi was combined with adoptive NK cell transfer [39]. Our data suggest that APTO253 as a novel MICA-regulating small molecule represents an additional promising candidate for combination therapy with NK cell transplantation to treat AML patients. The anti-tumor effects of APTO253 in AML were related to the inhibition of MYC [26]. We observed that the treatment of AML cells either with HDACi or with APTO253 resulted in a dose-dependent decrease of MYC levels and we demonstrated that cells resistant to MYC inhibition in response to araC-mediated DNA damage failed to upregulate MICA. This means that possibly both, the induction of KLF4 and the downregulation of MYC contribute to the APTO253-mediated induction of NKG2D-L. This is in line with the reported MYC-dependent suppression of *MICA* transcription [40]. Moreover, the anti-tumor activity of APTO253 was attributed to the induction of KLF4, which is often down-regulated in hematological cancers [41].

So far, APTO253 monotherapy trials to treat AML patients showed only a limited response with no severe side effects [42, 43]. Here, we unravel for the first time that the KLF4-inducer APTO253 re-activates MICA expression in AML cells. This provides a novel and unexpected link of APTO253 to immune cell activation and the NKG2D/NKG2D-L axis. Interestingly, the induction upon treatment with APTO253 appeared not to be limited to MICA, since also the NKG2D ligands MICB and ULBP1 were induced in an APTO253-dependent manner. A trend for induced expression was observed for ULBP3 (Supplementary Figure S6A), in line with the presence of KLF4 binding motifs in the respective gene regulatory regions. However, upregulation of ULBP2 which also harbors KLF4 binding motifs (Supplementary Figure S6B) was not affected meaning that the presence of a binding motif does not necessarily indicate a functional role. This was also shown for heat shock factor 1 (HSF1) binding motifs which are present in MICA/B and some of the ULBP genes. Of note, heat shock induces HSF1 promoter binding and expression of MICA/B, but there is no evidence for heat shock-induced ULBP expression [3]. This mirrors the diversity in the regulatory sequences in NKG2D ligand gene promoters and the heterogeneous expression in different cell types, although some partially common upstream mechanisms such as cellular stress signals or HDACi may exist. Taken together these results suggest that the combination of APTO253 with adoptive NK cell transfer may be beneficial for AML patients.

## Supplementary Information

Additional file 1. 

Additional file 2. 

## Data Availability

The datasets supporting the conclusions of this article are included within the article and its additional files.
