# Supplementary material for: KLF4-mediated upregulation of the NKG2D ligand MICA in acute myeloid leukemia: a novel therapeutic target identified by enChIP
Source: Cell Commun Signal. 2023 May 4;21:94. doi: 10.1186/s12964-023-01118-z (PMC10157933; doi:10.1186/s12964-023-01118-z)
Supplement: Supplementary file 2 — Additional file 1. [file 12964_2023_1118_MOESM1_ESM.zip › Legends Supplememtary Figures_ESM.docx]

Legends Supplementary Figures

**Fig. S1A**

**Transfection of HEK293 cells with the CRISPR/dCas9 system did not interfere with the induction of *MICA* transcription under LBH589 treatment.** HEK293 cells were transfected with pEF1a-FBdCas9 plasmid and gRNAs targeting the MICA promoter region. 48 h post-transfection cells were treated with 100 nM LBH589 for 4 h. Then, cells were subjected to RT-qPCR to measure *MICA* transcripts.

**Figure S1B:**

**Published ChIP data** on selected candidate factors (https://chip-atlas.org) demonstrating binding to the *MICA* promoter region.

**Figure S2:**

**Western Blot to detect KLF4 protein** in lysates of HEK293 cells upon transfection with different KLF4 expression constructs. An empty expression vector (ctrl) was used a control and a KLF4 expression vector was used as a positive control right lane).

**Figure S3:**

**Western Blot to detect KLF4** protein upon vehicle (ctrl) and Kenpaullone (ken) treatment.

**Figure S4:**

**LBH589 suppresses the expression of *c-MYC* in AML cell lines.** AML cell lines were treated with 100 nM LBH589 for 4 h. RT-qPCR analysis was performed to detect the mRNA level of *c-MYC* in HL60, NB4, and MM6 cells. Data show the mean (±SEM) of three biological replicates. Significance was calculated using an unpaired *t-*test. *****p* ˂ 0.0001.

**Figure S5:**

**APTO253 did not induce the expression of *MICA* and *KLF4* in lymphocytes of healthy donors.** Lymphocytes were isolated from healthy donors and treated with APTO253 as indicated for 18 h. RT-qPCR analysis was performed to detect the mRNA level of *MICA* and *KLF4.* Data show the mean (±SEM) of three biological replicates. Significance was calculated using an unpaired *t-*test. **p* ˂ 0.05, ***p* ˂ 0.01, ****p* ˂ 0.001.

**Figure S6A:**

**APTO253 induces the expression of NKG2D ligands in HL60 cell line.** HL60 cells were treated with APTO253 at concentration of 300 nM for 18 h. RT-qPCR analysis was performed to detect the mRNA level of **a.** *MICB* and **b.** *ULBP1* and **c*.*** *ULBP3****.*** Data show the mean (±SEM) of three biological replicates. Significance was calculated using an unpaired *t-*test. **p* ˂ 0.05.

**Figure S6B:**

The Integrative Genomics Viewer (IGV) Software was used to determine the ChIP-seq data for KLF4 in the promoters of MICA, MICB and ULBP1-3. The red box indicates the enrichment of KLF4 in the NKG2D ligand promoter regions. In the case of MICA, the region was isolated using the enChIP approach. ChIP-Seq was performed with primary human pulmonary artery endothelial cells (PAEC) transduced with caMEK5 compared with GFP controls. (doi: [10.1038/s41467-022-32566-9](http://dx.doi.org/10.1038/s41467-022-32566-9).). caMEK5: constitutively activated MEK5.
