# Supplementary figures and images for: KLF4-mediated upregulation of the NKG2D ligand MICA in acute myeloid leukemia: a novel therapeutic target identified by enChIP
Source: Cell Commun Signal. 2023 May 4;21:94. doi: 10.1186/s12964-023-01118-z (PMC10157933; doi:10.1186/s12964-023-01118-z)

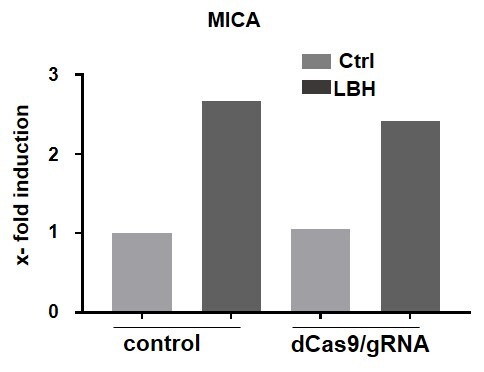

Supplement: Supplementary file 2 — Additional file 1. [file 12964_2023_1118_MOESM1_ESM.zip › supp-1A.jpg]

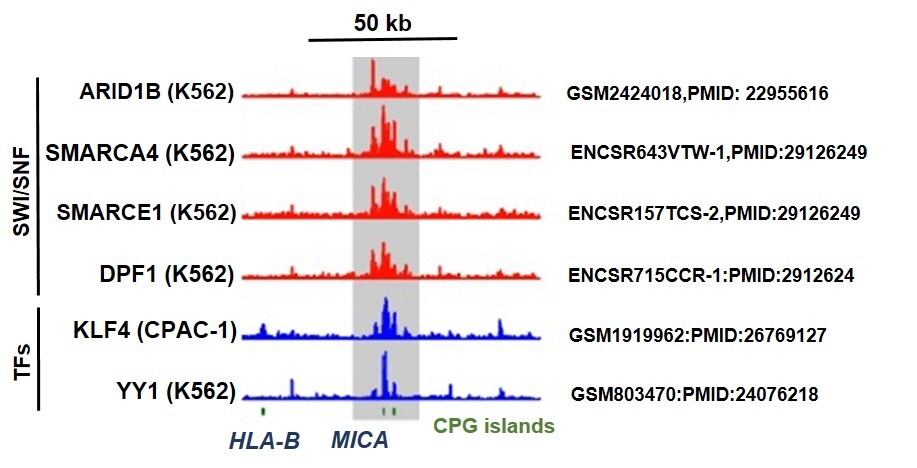

Supplement: Supplementary file 2 — Additional file 1. [file 12964_2023_1118_MOESM1_ESM.zip › supp-1B.jpg]

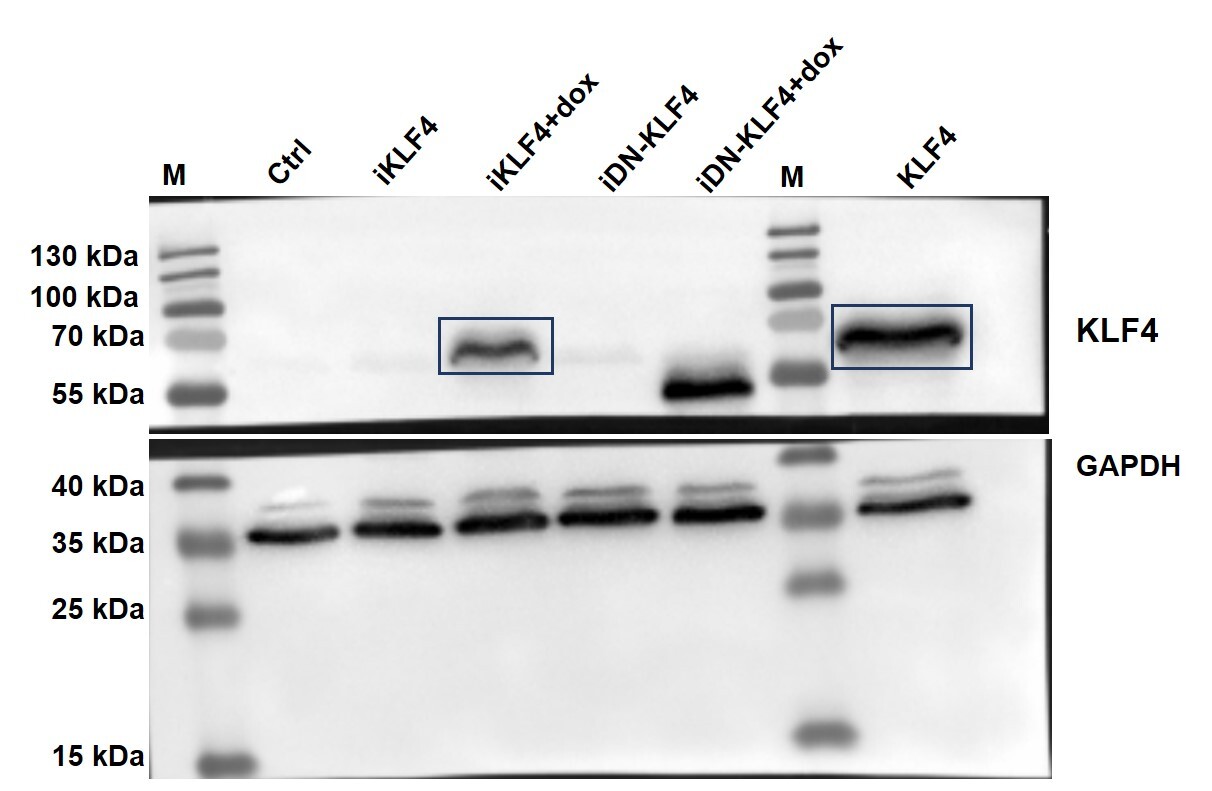

Supplement: Supplementary file 2 — Additional file 1. [file 12964_2023_1118_MOESM1_ESM.zip › supp-2.jpg]

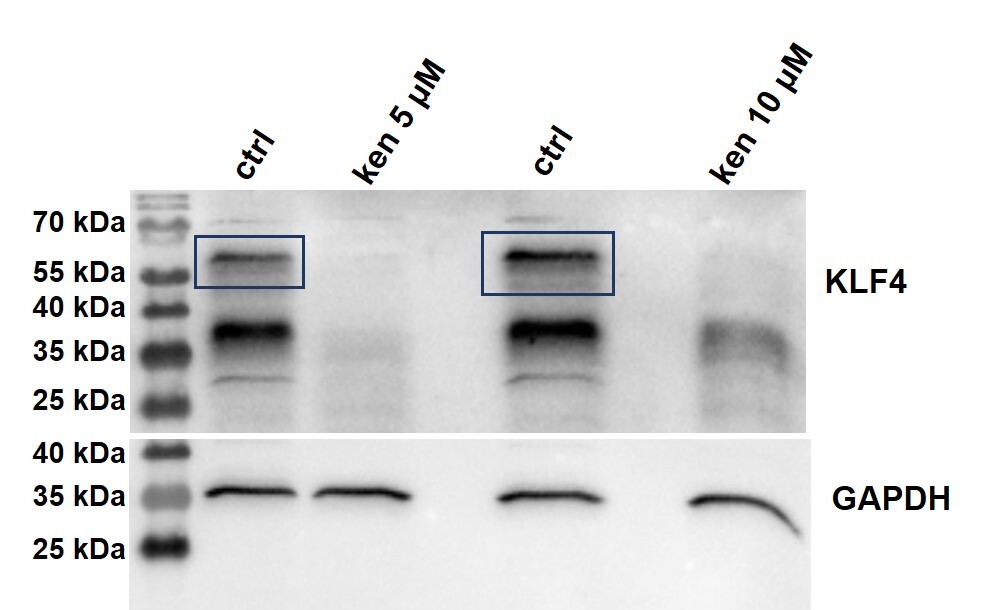

Supplement: Supplementary file 2 — Additional file 1. [file 12964_2023_1118_MOESM1_ESM.zip › supp-3.jpg]

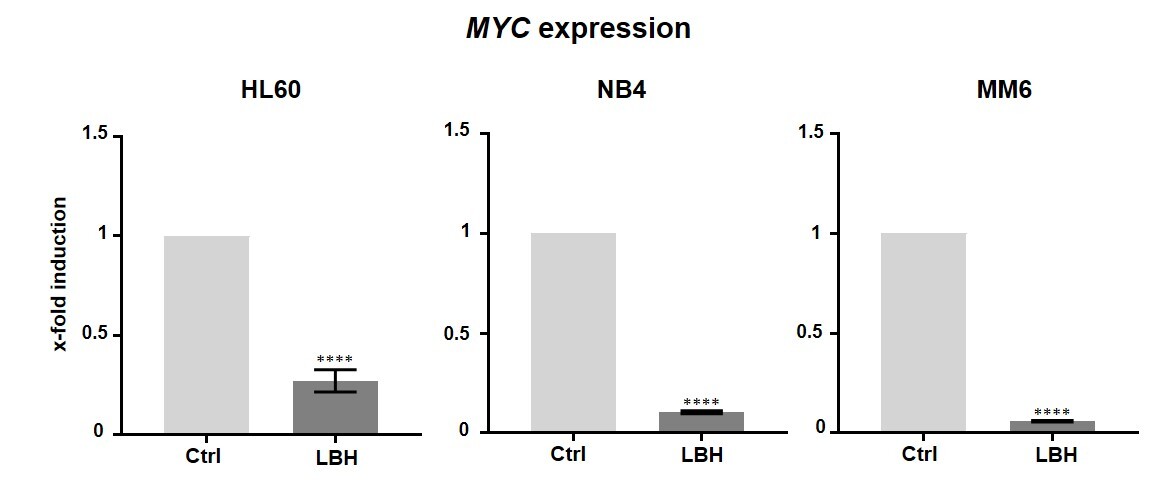

Supplement: Supplementary file 2 — Additional file 1. [file 12964_2023_1118_MOESM1_ESM.zip › supp-4.jpg]

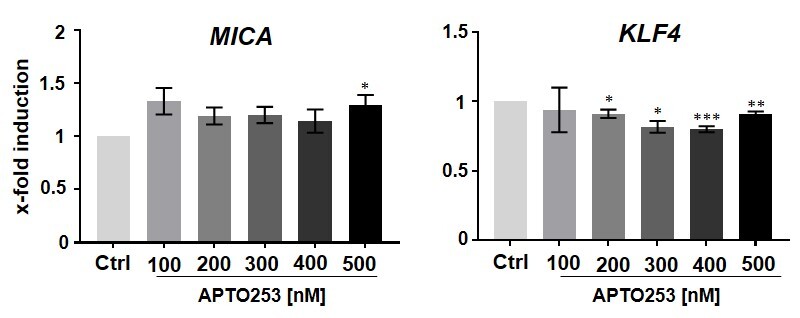

Supplement: Supplementary file 2 — Additional file 1. [file 12964_2023_1118_MOESM1_ESM.zip › supp-5.jpg]

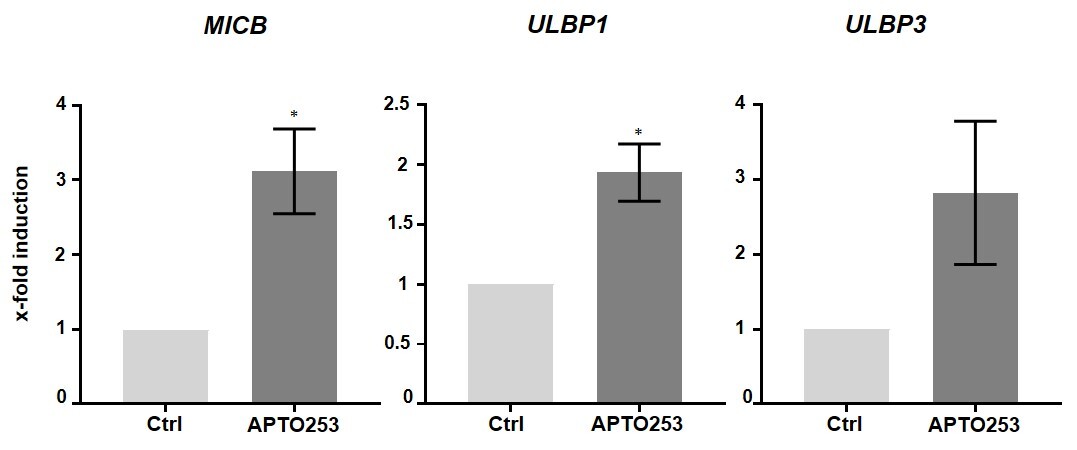

Supplement: Supplementary file 2 — Additional file 1. [file 12964_2023_1118_MOESM1_ESM.zip › supp-6A.jpg]

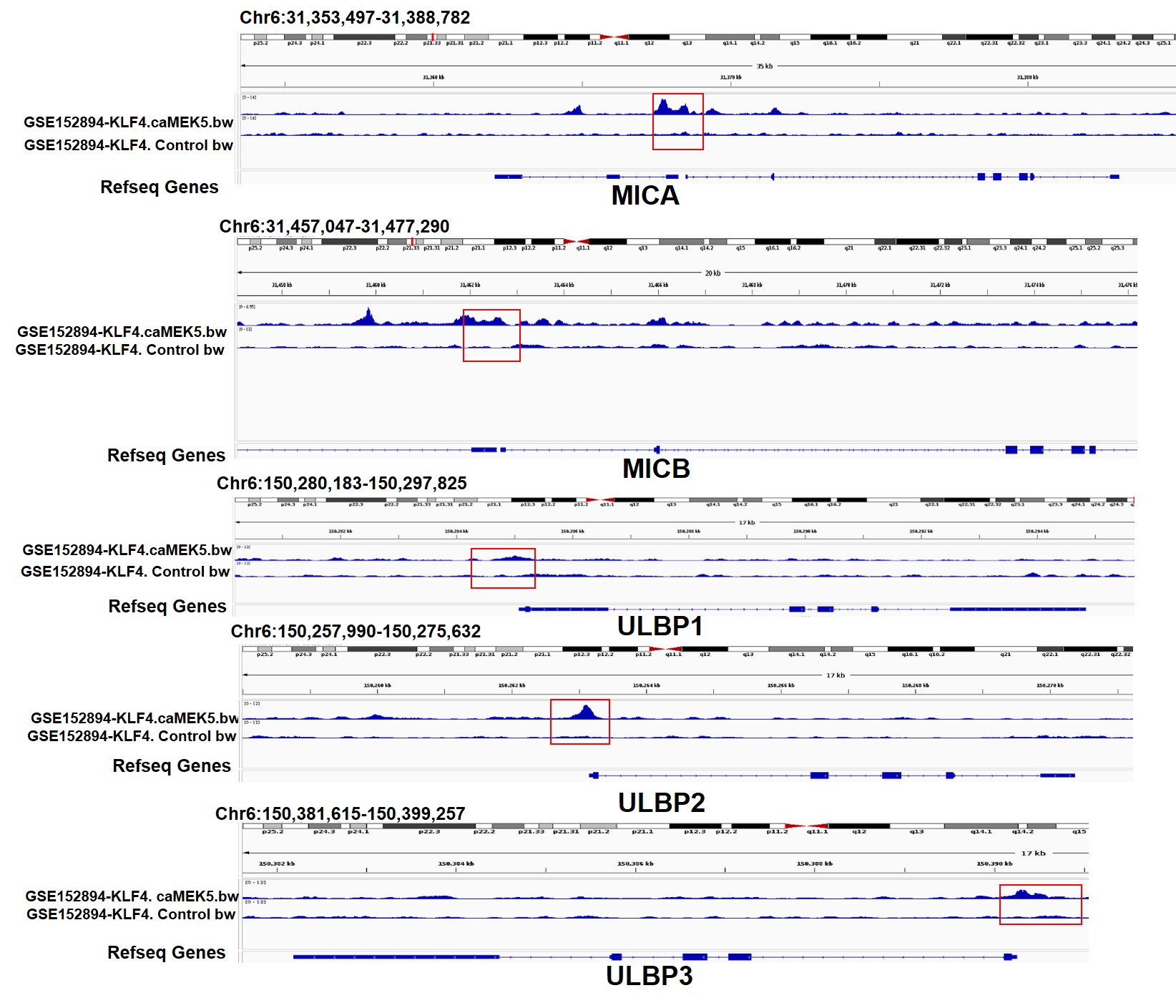

Supplement: Supplementary file 2 — Additional file 1. [file 12964_2023_1118_MOESM1_ESM.zip › supp-6B.jpg]
